# Isolated Sphenoid Sinus Inflammatory Disease-A Report of 14 Cases

**DOI:** 10.22038/ijorl.2019.39416.2304

**Published:** 2020-03

**Authors:** Gian-Luca Fadda, Anna D’Eramo, Alessandro Grosso, Andrea Galizia, Giovanni Cavallo

**Affiliations:** 1 *Department of Otolaryngology, San Luigi Gonzaga Hospital, University of Turin, Orbassano, Italy.*

**Keywords:** Cavernous sinus, Isolated sphenoid sinus inflammatory diseases, Sphenoid sinusitis, Visual disturbance

## Abstract

**Introduction:**

Isolated sphenoid sinus inflammatory diseases (ISSIDs) are responsible for about 75% of isolated sphenoid sinus opacifications. Computer tomography (CT) and magnetic resonance imaging (MRI) should be used in a complementary manner for the assessment of ISSIDs. This evaluation sheds some light on the extent of disease and intracranial and intra-orbital involvement.

**Materials and Methods:**

The current study aimed to evaluate the medication histories of 14 patients who underwent endoscopic sinus surgery (ESS) for ISSIDs within 2015-2018. This assessment was carried out to analyze the presenting symptoms, diagnostic work-up, additional therapies, and complications. Moreover, it can help us compare our data with pertinent literature.

**Results:**

As evidenced by the obtained results, ISSID lesions included bacterial sphenoiditis (42.9%), fungus ball (21.4%), invasive fungal sphenoiditis (14.3%), mucocele (14.3%), and retention cysts (7.1%). In addition, headache was found to be the major complaint, followed by nasal symptoms. Diplopia, and signs and symptoms of the involvement of other cranial nerves were less frequent. All patients underwent endoscopic transnasal sphenoidectomy. The overall survival rate was reported as 92.9% (13/14), and all patients with cranial nerve palsies demonstrated complete clinical remission.

**Conclusion:**

Both the review of related literature and our clinical cases were indicative of the dangerous consequences of ISSIDs. Their varied and unspecific presentation and the limited reliability of nasal endoscopy required the cooperation of ENT (ear, nose, and throat) team with other specialists to make an accurate diagnosis and decide on the most appropriate therapeutic choices. If the signs of intracranial complications were detected, surgery should be promptly performed to maximize the chances of recovery.

## Introduction

Isolated sphenoid sinus inflammatory diseases (ISSIDs) are responsible for about 75% of isolated sphenoid sinus opacifications (1). They include acute and bacterial sphenoid sinusitis, fungal rhinosinusitis (FRS), and mucocele (2).

The sphenoid sinus has an intimate anatomic relationship with the cavernous sinus (CS), pituitary gland, internal carotid artery, cranial nerves (CNs) (II,III,IV,V1-V2,VI), the sphenopalatine ganglion, sphenopalatine artery, the pterygoid canal and its nerve, vessels, and dura mater (3-6). Therefore, the spread of infection or inflammation beyond the sphenoid sinus to these neighboring structures may result in serious or even fatal intracranial and orbital complications. In contrast to non-invasive FRS, mortality is reported to be as high as 40-80% in acute invasive fungal disease with orbit or skull base involvement (7-9). The diagnosis and treatment of ISSIDs are inappropriately delayed due to its subtle and non-specific signs and symptoms. Headache is the most common presenting symptom (70-100%) (5,10). On the other hand, cranial nerve involvement is a less common, but not exclusively, rare complication of ISSIDs (6-12%).

Nonetheless, sometimes it is the prominent or sole clinical feature at diagnosis (5). Imaging studies, such as computed tomography (CT) and magnetic resonance imaging (MRI), are considered the gold standard to formulate a diagnostic hypothesis and identify possible complications. Therefore, we presented our retrospective analysis of 14 ISSID cases and reported on the clinical features, complications, and the diagnostic and therapeutic approach. A review of the literature was also carried out.

## Materials and Methods

A number of 14 patients presenting with headache with or without cranial nerve palsy or intracranial complications were diagnosed with ISSIDs at the Otolaryngology Department of the University of Turin, San Luigi Gonzaga Hospital within April 2015-April 2018.They were diagnosed and treated both medically and surgically at our institute. 

It is noteworthy that we did not include the patients whose sphenoid sinusitis was associated with the inflammation of other sinuses or those with sphenoid involvement by benign or malignant tumors.All patients underwent a physical examination, followed by endoscopy; moreover, a CT scan was performed in all patients. Nonetheless, MRI was administered when visual changes were clinically evident or fungal invasive infections were suspected due to the erosion of the adjacent orbital bone and cranial cavities. Although CT scan and MRI can suggest a diagnosis, an accurate diagnosis was only established after cultural or histological confirmation of the operative specimens. Patients were followed postoperatively by endoscopic examination for at least 8 months for inflammatory diseases and fungus ball. Moreover, patients with invasive fungal sinusitis were followed for a longer time period and underwent a radiological follow-up with MRI. 

Patient demographics, clinical manifestations, cranial nerve, cavernous sinus or brain involvement, as well as radiologic findings, treatment, culture, and pathological reports were evaluated. Based on a systematic literature review, we identified 10 major studies conducted on 891 patients using the term “isolated sphenoid sinus disease” on PubMed.

## Results

Based on literature review, ISSIDs accounted for approximately 71% ranging within 60.6% to 95% (1,4,5,10-16). The current study was performed on a number of 14 patients (4 males and 10 females) within the age range of 18-83 years with a mean age of 46.9 years (SD±19.8). ISSID lesions included bacterial sphenoiditis (6/14,42.9%), fungus ball of sphenoid sinus (3/14,21.4%), invasive fungal sphenoiditis (IFS) in immunocompromised hosts (2/14,14.3%), retention cysts (1/14,7.1%), and mucocele (2/14,14.3%) (Table.1) (^Fig’s 1^-4).

**Fig 1:**
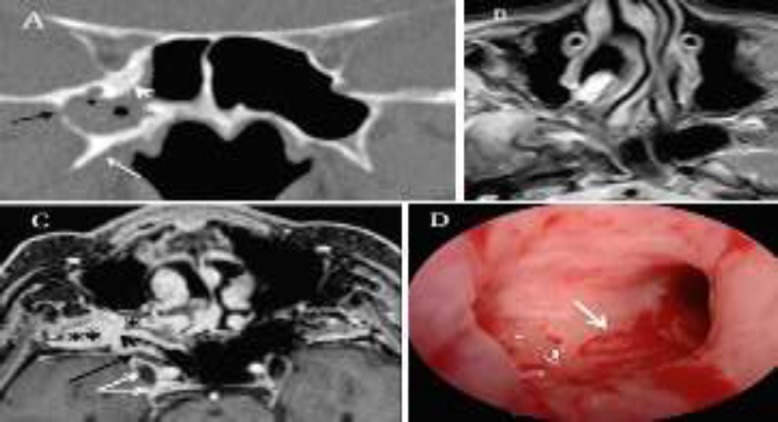
(A) Coronal CT scan shows an opacity of the lateral recess of the right sphenoid sinus (SS) with thickening and sclerosis of bone surrounding the pterygoid process (white arrow) and the orbital apex (white arrowhead). Focal interruption of the foramen rotundum (small star) and lateral recess of the sphenoid sinus is also present.(B) Axial T2-weighted MRI demonstrates a marked hypointense signal (small star) in the lateral recess of the SS with inflamed mucosa at the periphery. (C) Axial gadolinium enhanced T1-weighted MRI shows soft tissue protruding through the breach in the lateral recess wall of the SS (black arrowhead), invading the right internal pterygoid muscles, the masticatory fossa (double small star), and the pterygopalatine fossa (single small star) and infiltrating CN V2 (black arrow) and CN V3 (white arrow) trigeminal branches. Endocranially, the tissue was extended into the cavernous sinus surrounding the Gasser ganglion (double angled arrow). (D) Intraoperative view during endoscopic sinus surgery indicates the erosion of the lateral recess wall of the right SS (white arrow)

**Fig 2:**
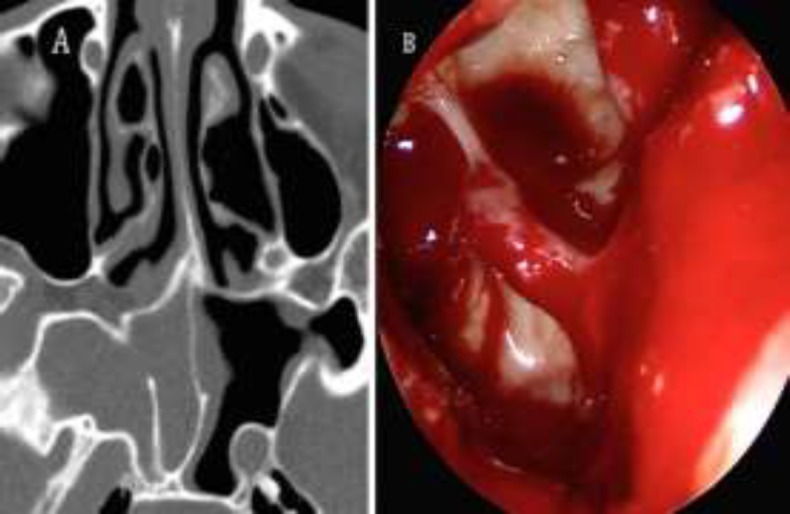
(A) Axial CT scan displays a complete opacification of the right sphenoid sinus, (B) hyperpneumatization of the pterygoid recess, and corresponding endoscopic endonasal surgery image

**Fig 3:**
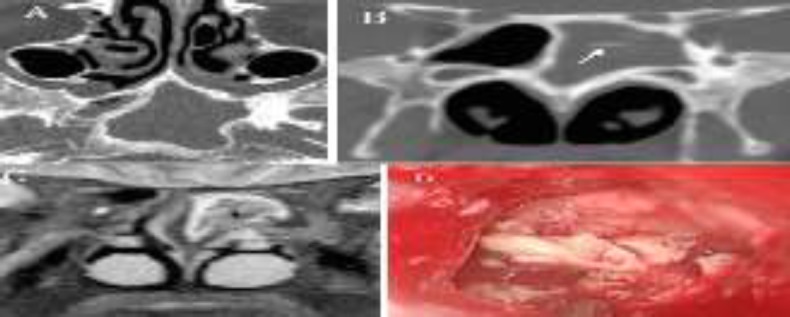
Axial (A) and coronal (B) CT scan demonstrates the opacification of the left sphenoid sinus with calcifications (white arrows) and marked thickening of the walls of the sinus. (C) Coronal T2-weighted MRI indicates an isointense signal from the fungal mass (asterisk) with hyperintense inflamed mucosa at the periphery of the sinus. Intraoperative view of the fungal concrement in the sphenoid sinus floor (D)

**Fig4:**
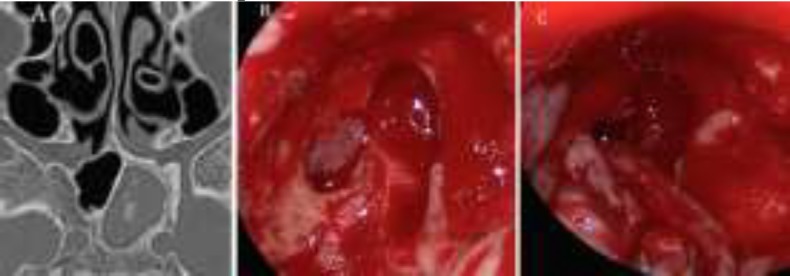
(A) Axial CT scan displays heterogeneous opacification in the left sphenoid sinus with remodeling of the adjacent bone. (B) Intraoperative images demonstrate mucocele after the opening of the anterior wall of the sphenoid sinus. (C) Endoscopic removal of the mucocele

**Table 1:** Clinical information for 14 patients with complicated sphenoid sinusitis in the present study: L=left; R=right; CS=cavernous sinus; SER=sphenoethmoidal recess; CD=cardiovascular diseases; CI=cerebellar ischemia; ICA=internal carotid artery; I=histopathological finding; M=microbiological finding; * =immunocompromised patient

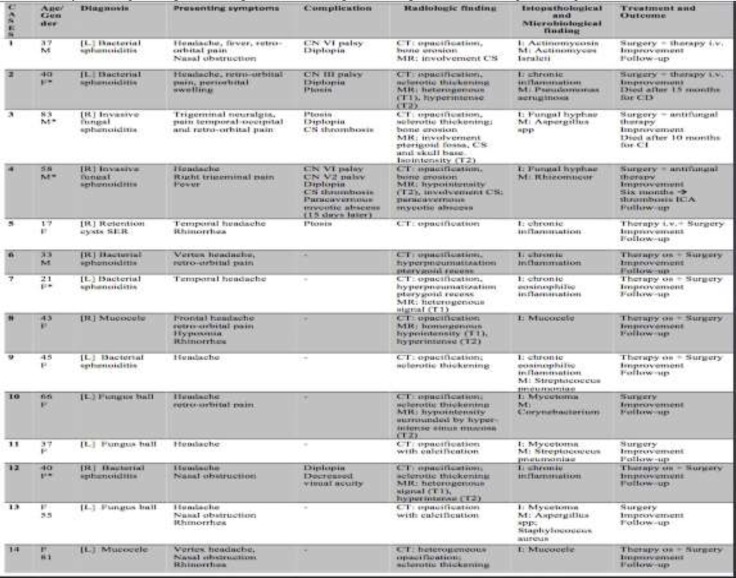

Notably, the symptomatology was often unspecific. As in most series, headache was the most frequent presenting complaint (13/14, 92.9%). Other clinical features at presentation included retro-orbital pain (6/14,42.9%) and nasal symptoms (rhinorrhea, nasal obstruction, and hyposmia) (6/14,42.9%). The most common complication was reported to be diplopia (5/14, 35.7%), followed by ptosis (3/14,21.4%), cranial nerve (CN) VI palsy (2/14,14.3%), CS involvement (2/14,14.3%), CN V2 palsy (1/14,7.1%), CN III palsy (1/14,7.1%), and decreased visual acuity (1/14,7.1%). In addition, one patient had multiple CN palsies involving CN VI and V2 unilaterally.Nasal endoscopy revealed mucosal edema or mucopurulent discharge into the sphenoethmoidal recess in 5/14 patients. We believe that the absence of alterations in endoscopic examination should not rule out sphenoid disease since abnormal endoscopic findings were detected in 35.7% of our sample which was consistent with other series (1,5,17,18). 

All patients underwent a CT scan and complete opacification of the involved sphenoid sinus was the most common finding (13/14,92.9%) It was followed by sclerotic thickening of the sphenoidal walls (6/14, 42.9%), calcification suggesting fungus ball (4/14,28.6%), and bony erosion of the lateral wall (3/14,21.4%). Moreover, hyperpneumatization of the pterygoid recess was observed in two cases (#6 and #7). MRI was performed in eight patients (57.1%) when fungal invasive infections were suspected or at the event of clinically evident involvement of CNs, such as ophthalmoplegia and diplopia, as well as CT evidence of bone erosion of the sphenoid sinus wall. Under general anesthesia, all patients underwent endoscopic transnasal sphenoidotomy to drain or remove the sinus inflammatory pathology. In nine cases (64.3%), a concha bullosa was treated with surgical plastic. All patients received concurrent antibiotics and additional antifungal drugs after the surgery when the operative specimen was positive for invasive fungal sinusitis. Six patients (42.9%) presented with clinically evident CN involvement; therefore, they required surgical drainage or debridement of the affected sphenoid sinus. These procedures were carried out within 2 days from the first observation and a positive culture was obtained in seven patients (50%).

The overall survival rate was reported as 92.9% (13/14), and all patients with CN palsies demonstrated complete clinical remission. Both patients with IFS developed post-surgical complications. One of them (#3) died of bilateral cerebellar ischemia which was not related to sinus pathology 11 months after the surgery. On the other hand, an 18-mm paracavernous mycotic abscess was detected in the other patient (#4) at routine angio-MRI follow-up 15 days after the surgery. With antifungal therapy, the abscess progressively shrank in 6 months.

## Discussion

Isolated sphenoid sinus disease is a rare pathology that accounts for 1.0-2.7% of all paranasal sinus conditions (12,19-24). The most commonly reported etiology of ISSIDs is bacterial sphenoiditis (within the range of 17.4-64%) (1,2,4,5,10,13-15,25), followed by sphenoid mucocele (in the range of 17.5-47.8%) (2, 4,5,10,13–15). 

ISSIDs are generally insidious with subtle and non-specific symptoms; therefore, diagnosis requires a high index of suspicion, careful endoscopic examination, and specific imaging. Headache has been reported in 64.3-100% of ISSID patients (1,5,10,13,25,27) with different localization, including retro-orbital, frontal, vertex, and diffuse (3,4,10,13,14,19,21). It has been accepted that headache alone is a non-specific symptom which might suggest, but not exclusively, indicate the presence of a sphenoidal pathology (28,29). Nevertheless, a highly intense headache, especially the ones which are poorly responsive or non-responsive to drugs, need prompt imaging, such as a brain CT scan, in immunocompromised patients (20). According to the current literature, the second most common presenting symptom was nasal obstruction which was possibly associated with purulent rhinorrhea and hyposmia (13,14,28).

Less frequently, diplopia due to cranial nerve palsy may be the initial presenting symptom of sphenoid sinusitis, as was the case with five patients (35.7%) in our sample. Diplopia in sphenoidal diseases is mostly caused by CN VI involvement due to its medial anatomical location in the CS and proximity to the sphenoid sinus; however, it can be accompanied by other cranial palsies (2,25,31,32). In their revision of 17 cases of ISSIDs, El Mograbi and Soudry revealed that diplopia could be associated with isolated CN VI palsy (76%) or isolated CN III palsy (18%)(25), while one patient (6%) presented multiple CN palsies involving nerves III, IV and VI unilaterally. Chen et al. (2) reported that 47.8% (11/23) of patients in their series suffered from diplopia: 5 due to CN III palsy and 6 owing to CN VI palsy. It is worthy to note that all patients recovered after surgery.

Cranial nerve palsy may result from direct nerve infiltration by the sphenoidal inflammatory process, compression by an expansive lesion (i.e.mucocele), vasculitis of the nervous sheath vessels or cavernous sinus thrombosis (CST) which causes an ischemic infarction of the cranial nerve (2,33-35). In rare cases, ISSIDs which are complicated by cranial neuropathy can develop into potentially life-threatening disorders, such as meningitis, cerebritis, CST, or internal carotid artery involvement (25). Microorganisms can spread directly from the mucosa in the presence of breached sphenoidal walls, or they can be conveyed to the CS via communicating veins or osteomyelitis of interposed diploic bone (36). Wang et al. (34) reported eight cases of CST secondary to sphenoid sinusitis with headache and one or more ophthalmologic symptoms or signs. Nasal endoscopy can be useful in the diagnosis of ISSIDs; in fact, some authors have found endoscopic anomalies in 65-76% of ISSDs (10,12,37). CT scan is an excellent tool in the diagnosis of ISSIDs in patients. It should be complemented with MRI in patients with expansive lesions, bone erosion, or sclerotic thickening of the sphenoidal walls, or when the involvement of intracranial and skull base structures, such as the CS, optic or cranial nerves, internal carotid artery, and cerebral tissue, cannot be ruled out (11,12,21,37,38).

Endoscopic sphenoidectomy should be promptly performed in patients with signs or symptoms of CN injury at the absence of no improvement after 24-48 h of intravenous antibiotics. Nonetheless, it should be performed immediately if fungal disease is suspected (3).

 It is noteworthy that endoscopic spheno- idectomy is the only way to ensure the removal of fungal debris and establish a diagnosis (13,14,39). In agreement with Castelnuovo et al. (14), we believe that the surgical approach to this sinus is a delicate procedure; consequently, it should only be performed by skilled surgeons with adequate surgical instrumentation.

The consensus on the treatment of septic CST includes intravenous antibiotics and surgical drainage of the sphenoid sinus which should be promptly performed, while surgical intrusion into the CS is difficult and not recommended (25,34,40,41). On the other hand, considerable controversy exists over the use of corticosteroids for CST. They can reduce inflammation resulting in an improvement in cranial nerve function and orbital congestion (40,42,43). Nonetheless, we agree with Chen et al. (2) in warning that steroids alone may facilitate the spread of infection, especially in acute fungal sinusitis. In addition, some studies have reported a significant reduction in morbidity and mortality when anticoagulants were added at the initiation of treatment (42,44).

## Conclusion

Isolated sphenoid sinus pathology is known as an uncommon clinical condition. It frequently presents with intense and refractory headaches and sometimes with diplopia which could be due to cranial nerve palsy or cavernous sinus thrombosis. Radiology is essential in the diagnosis and assessment of ISSIDs since the symptoms are often generic and unspecific. In ISSIDs, prompt diagnosis and early surgical intervention are crucial to prevent complications or allow for complete recovery of ocular function when it is impaired.

Otolaryngologists play a significant role in the management of such a complex and life-threatening condition that involves many different fields of expertise. They can orchestrate multiple specialists and gain direct access to the affected area, thereby taking the initial and most essential step towards accurate diagnosis and therapy.
